# Attenuation of atherosclerotic lesions in diabetic apolipoprotein E-deficient mice using gene silencing of macrophage migration inhibitory factor

**DOI:** 10.1111/jcmm.12521

**Published:** 2015-02-08

**Authors:** Hui Sun, XianJun Zhang, Lei Zhao, Xi Zhen, ShanYing Huang, ShaSha Wang, Hong He, ZiMo Liu, NaNa Xu, FaLin Yang, ZhongHua Qu, ZhiYong Ma, Cheng Zhang, Yun Zhang, Qin Hu

**Affiliations:** a Key Laboratory of Cardiovascular Remodeling and Function Research, Chinese Ministry of Education and Chinese Ministry of Health, Department of Cardiology, Shandong University Qilu HospitalJinan, Shandong, China; b Department of Dermatology, Shandong University Qilu HospitalJinan, Shandong, China; c Department of Clinical Laboratory, Shandong University Qilu HospitalJinan, Shandong, China

**Keywords:** macrophage migration inhibitory factor, diabetes mellitus, atherosclerosis, apolipoprotein E-deficient mice, inflammation

## Abstract

Macrophage migration inhibitory factor (MIF) involves the pathogenesis of atherosclerosis (AS) and increased plasma MIF levels in diabetes mellitus (DM) patients are associated with AS. Here, we have been suggested that MIF could be a critical contributor for the pathological process of diabetes-associated AS by using adenovirus-mediated RNA interference. First, streptozotocin (STZ)-induced diabetic animal model was constructed in 114 apolipoprotein E-deficient mice (apoE−/− mice) fed on a regular chow diet. Then, the animals were randomly divided into three groups: Adenovirus-mediated MIF interference (Ad-MIFi), Ad-enhanced green fluorescent protein (EGFP) and normal saline (NS) group (*n* ≈ 33/group). Non-diabetic apoE−/− mice (*n* = 35) were served as controls. Ad-MIFi, Ad-EGFP and NS were, respectively, injected into the tail vein of mice from Ad-MIFi, Ad-EGFP and NS group, which were injected repeatedly 4 weeks later. Physical, biochemical, morphological and molecular parameters were measured. The results showed that diabetic apoE−/− mice had significantly aggravated atherosclerotic lesions. MIF gene interference attenuated atherosclerotic lesions and stabilized atheromatous plaque, accompanied by the decreased macrophages and lipids deposition and inflammatory cytokines production, improved glucose intolerance and plasma cholesterol level, the decreased ratio of matrix matalloproteinase-2/tissue inhibitor of metalloproteinase-1 and plaque instability index. An increased expression of MIF and its ligand CD74 was also detected in the diabetic patients with coronary artery disease. The results suggest that MIF gene interference is able to inhibit atherosclerotic lesions and increase plaque stability in diabetic apoE−/−mice. MIF inhibition could be a novel and promising approach to the treatment of DM-associated AS.

## Introduction

Diabetes mellitus (DM) is a systemic disease affecting both the quality and length of life. It is well-established that atherosclerosis is a major complication of both type 1 and type 2 DM 1,2. Indeed, up to 75% of patients diagnosed with diabetes ultimately died from atherosclerosis-related cardiovascular diseases such as myocardial infarction (MI), peripheral artery disease and stroke 3,4. DM patient with coronary artery disease (CAD) has thus become a major public health concern 5. However, the mechanisms whereby diabetes accelerates cardiovascular disease are still unclear. Recent studies have identified that atherosclerosis with DM has low-grade inflammation which can stimulate macrophage-foam cell formation 6,7.

Macrophages migration inhibitory factor (MIF), as a pleiotropic cytokine, plays a critical role in several inflammatory conditions including various tumours, atherosclerosis, diabetes and obesity in both animal and human 7–11. The pro-inflammatory effects of MIF may be related to the products of cytokines like tumour necrosis factor-α (TNF-α) and interleukin (IL)-6 12. MIF also influences glucose metabolism at several levels, affecting both insulin production in the pancreatic beta cell and the cells targeted by insulin 7. Atsumi *et al*. 13 have confirmed that MIF could regulate glucose metabolism by increasing glucose uptake in peripheral tissue that is related to the cytokine like TNF-α. Moreover, MIF plays a key role in glucose homoeostasis during periods of stress and in the development of type 1 and type 2 DM. Direct clinical evidence reveals that a higher plasma level of MIF is found in patients with impaired glucose tolerance or type 2 DM 14. Pan *et al*. 15 have found that the MIF deficiency could impair atherosclerosis in LDLR-deficient mice through the reduction in plaque area, lipid and macrophages. Moreover, using the neutralizing anti-MIF monoclonal antibody to block the MIF could reduce intimal macrophage content as well as the circulating and local aortic inflammatory mediators 16. The development of atherosclerosis and the plaque vulnerability are strongly controlled by the recruitment of leucocytes and their expression of pro-inflammatory cytokines like TNF-α and monocyte chemotactic protein-1 (MCP-1) 12,17–20. These existing data suggest that MIF is induced by lipid, which is the main instigator of atheroma lesion formation, and in turn regulates key events in lesion formation, inflammatory cell activation, plaque instability and neointimal responses 9. However, to date, the link between MIF and diabetes-associated atherosclerosis has not been established. We have been suggested that MIF could be a critical contributor for the pathological process of diabetes-associated atherosclerosis. In this study, we investigated the effect and its potential mechanism of MIF on STZ-induced diabetes-associated atherosclerosis by using adenovirus-mediated MIF gene interference in mice.

## Materials and methods

### Recombinant adenovirus

First, 4 miR sequences (named A, B, C and D; Table S1) targeting mouse *MIF* gene (NM_010798.2) were designed and constructed, which exhibited 79%, 76%, 68% and 63% reduction, respectively. After RNAi screening, the most effective miR sequences (named A) was cloned generated. Then recombinant miR-MIF adenovirus was generated and purified (Ad-MIFi). Ad-enhanced green fluorescent protein (EGFP) viral suspension was obtained from Invitrogen, Shanghai, China.

### Animal model and Gene transfer

**Fig 1:**
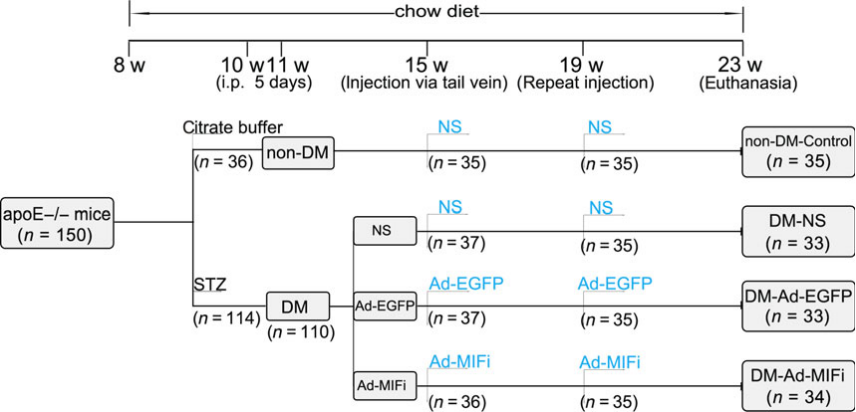
Flow chart showing the animal grouping and time line of the experimental protocol *in vivo*. STZ, streptozotocin; Ad-MIFi, adenovirus-mediated macrophage migration inhibitory factor gene interference; Ad-EGFP, adenovirus-mediated enhanced green fluorescent protein; NS, normal saline; i.p., intraperitoneal injection; w, week old; d, day.

150 ApoE −/− mice (male, 8 weeks old) were randomly divided into five per cage with access to standard mouse chow diet (5% fat, 0.02% cholesterol with no cholic acid) for 15 weeks until sacrifice. The animal grouping and time line of the experimental protocol were shown in Figure1. As previous described 9,21, diabetic apoE−/− mice were constructed by intraperitoneal injecting of streptozotocin (STZ) (at dose of 45 mg/kg/day, Boehringer, Mannheim, Germany) diluted by citrate buffer (pH 4.5, final concentration: 1%) for 5 days. ApoE−/− mice (*n* = 36) injected with vehicle were served as non-DM control. Only the mice with continuous blood glucose levels >15 mmol/l were recruited to the DM group (*n* = 110). These diabetic mice were then randomly divided into three groups: Ad-MIFi (*n* = 36), Ad-EGFP (*n* = 37) and normal saline (NS) (*n* = 37) groups. Ad-MIFi or Ad-EGFP diluted to a total volume of 100 μl was, respectively, injected into the tail vein of each mouse in two intervention DM groups, while the mice injected with normal saline served as vehicle controls. The adenovirus injection repeated 4 weeks later. All mice were killed 8 weeks later for further study. Although 150 mice were initially adopted onto the study, 15 mice had to be killed because of infection and death, but the study was still sufficiently powered to gain significant differences among the groups. All the experimental procedures were performed in accordance with the institutional guidelines of Qilu Hospital of Shandong University and were approved by Animal Care and Use Committee of Shandong University. The present investigations comply with the Guide for the Care and Use of Laboratory Animals published by the U.S. National Institutes of Health (NIH Publication No. 85-23, revised 1996).

### Biochemical assays

Serum total cholesterol (TC), triglycerides (TG), low-density lipoprotein cholesterol (LDL-C), high-density lipoprotein cholesterol (HDL-C) and glucose concentrations were measured by enzymatic assay using an automatic biochemical analyzer (ROCHE COBAS INTEGRA 800, Basel, Switzerland) from the fasting mice at the time-points dictated.

Three days before euthanasia, intraperitoneal Glucose Tolerance Test (ipGTT) was performed on 6 hrs fasted mice. The mice (*n* = 6 each group) were intraperitoneally injected with glucose (2 g/kg bodyweight, 20% glucose solution). Blood samples were respectively obtained from tail vein, at 0, 10, 30, 60, 90 and 120 min. after the glucose load, and blood glucose concentration was measured using a One Touch Ultra glucometer (Johnson&Johnson New Jersey, USA). The area under the curve (AUC) was then determined using the linear method of the trapezoid rule 21,22.

### ELISA

Blood MIF and IL-6 levels were measured using a commercially available ELISA kit (MIF Kit: EIAab, E0698m, Wuhan, China; IL6 Kit: eBioscience, BMS603/2, San Diego, CA, USA) according to the instructions.

### Tissue preparation, histological and immunohistochemical analysis

After the mice were anaesthetized, open the chest and expose the heart. The heart containing the aortic root, together with the artery from aortic arch to left and right common iliac artery was rapidly fixed and harvested at 4°C in phosphate-buffered 4% paraformaldehyde, pH 7.0–7.4. The whole length of the artery was fixed in 4% paraformaldehyde for measurement of the surface area covered by lipid-staining lesions. The frozen cross-sections of the aortic sinus embedded in freezing microtome (Leica CM1950, Nussloch, Germany), while the paraffin cross-sections of the aortic sinus (*n* = 8 for each group) embedded in paraffin wax sectioned by semi-automated rotary microtome (Leica, RM2245), which were mounted on slides for histological and immunohistochemical staining. Oil red O, haematoxylin and eosin, Masson trichrome and Picrosirus red were purchased from Sigma-Aldrich, Shanghai, China. Immunohistochemical staining for SMC: a rabbit antimouse α-SMA antibody (ab5694; 1:200, Abcam, Cambridge, UK); MAC387: rat antimouse monoclonal macrophages (ab22506; 1:250, Abcam, tested in the frozen sections); Endomucin: a rat antimouse Endomucin (eBioV.7C7; 1:150, eBioscience, San Diego, CA, USA); MIF: rabbit polyclonal IgG (sc-20121; 1:50, Santa Cruz Biotechnology, Dallas, TX, USA); CD74: goat polyclonal IgG (sc-5438; 1:50, Santa Cruz Biotechnology); MCP-1: rabbit polyclone antimouse CCL2/MCP-1 (BA1255; 1:200, BOSTER, Wuhan, China); tissue inhibitor of matrix metalloproteinase-1 (TIMP-1): matrix metalloproteinase-2 (MMP-2) (ab86607; 1:200, Abcam); CD4 (ab846, 1:200); VCAM1 (ab134047, 1:200); or ICAM1 (ab119871, 1:200) were also performed as previous described 23. Negative controls replaced primary antibody with non-immune IgG (Abcam). Quantified of the expression of these indexes by an automated image analysis system (Image-Pro Plus 6.0; Media Cybernetics, MD Rockville, USA) and the ratios of the positive staining area to the arterial plaque area represent their relative contents. Atherosclerotic plaque instability index 24 was calculated according to the standard plaque stabilization score formula: (Oil Red O^+^ area plus MAC^+^ area)/(α-SMA^+^ area plus collagen I^+^ area). For detrimental effect from STZ, haematoxylin and eosin staining of serial sections of liver tissue was performed after intraperitoneal injecting of low-dose STZ for 5 days. The tissue of remaining animals was perfused with PBS and snap frozen in liquid nitrogen and stored at −80°C for subsequent RNA and protein extraction.

### Quantitative real-time PCR

Real-time PCR was performed to determine the gene expression of MIF, CD74, MMP-9, MCP-1, TNF-α and Jun activation domain-binding protein 1 (Jab1) in atherosclerotic lesions by using SYBR Green Technology (Bio-Rad, California, USA), and the mouse housekeeping gene β-actin was applied as an internal control. The sequences of primers for β-actin or target gene sequence were mentioned on Table S2. The data were analysed by the 2^−ΔΔCT^ method. All experiments were repeated for at least three times.

### Western blot analysis

Western blot was performed to detect the protein levels of MIF, CD74, SMA, MAC and Collagen I. Membranes were respectively probed with the specific antibodies (MIF: 1:200; CD74: 1:300; SMA: 1:800; MAC: 1:800; Collagen: 1:400; Abcam, β-actin: 1:1000), followed by incubation with horseradish peroxidase-conjugated secondary antibodies (1:8000) and β-actin immunoblot analysis was applied to ensure equal sample loading. These primary antibodies of MIF, CD74, SMA, MAC and β-actin are the same as those in immunohistochemical analysis. The immunoreactive bands were visualized using enhanced chemiluminescent HRP Substrate (Millipore Corporation, Billerica, MA, USA).

### Immunohistochemistry for MIF and CD74 expression in patients with diabetes and CAD

Tissue samples of coronary arteries were obtained from autopsy of the CAD patients with DM (*n* = 5) and the patients with CAD alone (*n* = 5) and in compliance with institutional guidelines. There were three males and two females in each group. The mean age of patients was 50 years (range 38–65 years). In all cases, small lengths (0.5 cm) of left anterior descending (LAD) artery were dissected and immediately fixed in fresh 4% paraformaldehyde in 0.1 M PBS, pH 7.2, at 4°C for 2–4 hrs. Corresponding sections on separate slides were stained with haematoxylin and eosin, Masson's-trichrome and Sirius-red staining (for differentiation of collagen) and immunohistochemical staining for smooth muscle cells, macrophages, MIF and CD74, serially. All morphological analyses were performed on blinded slides. Similarly, quantified of the expression of these indexes by an automated image analysis system and the ratios of the positive staining area to the arterial plaque area represent their relative contents.

### Statistical analysis

A power analysis was performed for calculation of sample size. All data are presented as means ± SEM and were analysed by paired or one-way anova with *post hoc* analysis, as appropriate. A value of *P* < 0.05 was considered statistic significant. Data were analysed using SPSS 18.0 software (PASW, SPSS Inc., Chicago, IL, USA).

## Results

### STZ-induced diabetic apoE−/− mice and adenovirus transfection *in vivo*

**Fig 2:**
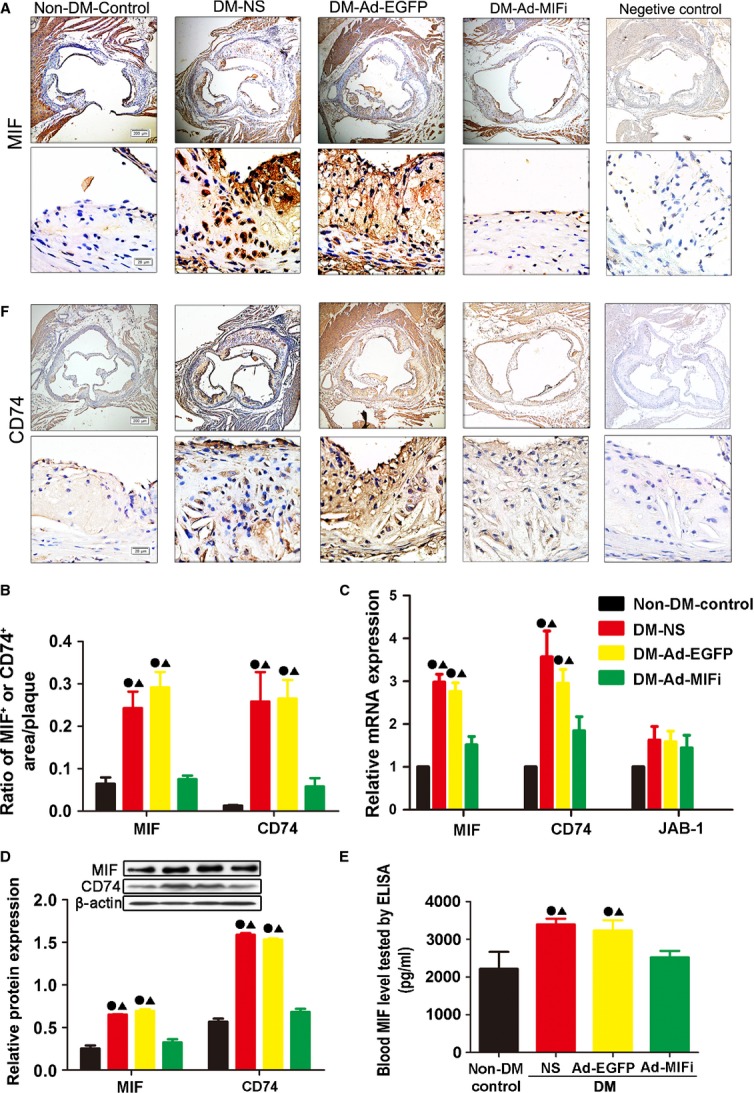
Efficiency of gene transfer in mice. (A and F) Representative images by immunohistochemical staining for MIF and CD74 (brown; top: scale bar = 200 μm, bottom: scale bar = 20 μm) in atheroslerotic lesions in the Ad-MIFi, Ad-EGFP, NS control and non-DM control groups. Negative controls replaced primary antibody with non-immune IgG (Abcam). (B) Quantitative analysis of the contents of MIF and CD74 by immunohistochemistry (*n* = 10). (C) Real-time PCR analysis of mRNA expression of MIF, CD74 and Jab-1 in the vessels. Relative expression was normalized to that of reference gene β-actin (*n* = 9). (D) Western blot analysis of the protein expression of MIF and CD74. Relative expression was normalized to that of reference β-actin (*n* = 8). (E) Plasma MIF levels tested by ELISA (*n* = 10). All quantitative data are means ± SEM. •*P* < 0.05 *versus* non-DM-Control group and ▴*P* < 0.05 *versus* DM-Ad-MIFi group.

After intraperitoneal injecting of low-dose STZ for 5 days, the slight cellular oedema, cytoplasm puffing and steatosis of hepatocyte was observed in apoE−/− mice (Fig. S1, see Data S1). Compared to non-diabetic controls, 14-week-old diabetic mice (3 weeks after STZ injection) had higher plasma cholesterol level and were hyperglycaemic (Table1). This hyperglycaemia lasted until the end of the study (12 weeks of diabetes). Similarly, blood TC of diabetic mice kept in higher level in comparison with the non-DM mice. The increased MIF contents in atherosclerotic lesions of diabetic apoE−/− mice were detected using immunohistochemistry, compared with non-DM control (Fig.2A and B). The increased MIF expression in DM group was further confirmed by RT-PCR and Western blot at both mRNA level and protein level (Fig.2C and D). In addition, the plasma MIF level was significantly increased in the DM mice (Fig.2E). As a ligand of MIF, CD74 protein and mRNA level was significantly up-regulated after STZ-induced hyperglycaemia, as detected by the immunohistochemistry, western blot and RT-PCR analysis (Fig.2B, C, D and F). As expected, the mRNA and protein levels of MIF were down-regulated after adenovirus-mediated MIF gene interference, as shown by immunohistochemistry, Western blot and RT-PCR analysis (Fig.2A–D). Moreover, MIF concentration in the serum was significantly reduced, while there were no differences between DM-NS group and DM-Ad-EGFP group (Fig.2E), suggesting the adenovirus itself did not influence the endogenous MIF expression. In the DM-Ad-MIFi group, CD74 protein and mRNA expression significantly reduced in comparison with the DM control group (Fig.2C and D). However, MIF gene interference had no impact on Jab1 mRNA expression (Fig.2C).

**Table 1:** Bodyweight and biochemical measurements in apoE−/− mice of the 4 groups

Parameters	Non-diabetes Control (*n* = 35)	Diabetes		
		Saline control (*n* = 33)	Ad-GFP (*n* = 33)	Ad-MIFi (*n* = 34)
Before i.p. STZ (9-week old)				
Bodyweight (g)	22.23 ± 0.63	22.12 ± 0.63	22.38 ± 0.44	22.37 ± 0.31
Glucose (mmol/l)	10.37 ± 0.56	10.41 ± 0.82	10.41 ± 0.85	10.10 ± 0.61
Serum lipid (mmol/l)				
CHO	8.82 ± 0.89	9.11 ± 1.22	7.57 ± 1.18	8.33 ± 1.92
TG	1.63 ± 0.25	1.71 ± 0.28	1.50 ± 0.21	1.25 ± 0.13
HDL-C	2.99 ± 0.43	3.65 ± 0.48	3.02 ± 0.50	3.54 ± 0.82
LDL-C	0.78 ± 0.10	0.77 ± 0.07	0.61 ± 0.11	0.68 ± 0.15
3 weeks after i.p. STZ/1-week before virus injection (14-week old)				
Bodyweight (g)	24.51 ± 0.51	22.15 ± 0.71*	21.3 ± 0.52*	21.2 ± 0.30*
Glucose (mmol/l)	10.84 ± 0.71	19.79 ± 2.35*	19.7 ± 1.86*	19.7 ± 1.12*
Serum lipid (mmol/l)				
CHO	8.85 ± 0.87	21.47 ± 3.58*	16.8 ± 1.57*	21.0 ± 2.93*
4 weeks after twice virus injection/after euthanasia (23-week old)				
Bodyweight (g)	25.07 ± 0.89	22.70 ± 0.41*	22.3 ± 0.61*	21.6 ± 0.39*
Glucose (mmol/l)	8.88 ± 0.71	20.39 ± 2.41*	25.7 ± 3.27*	10.5 ± 2.31*
Serum lipid (mmol/l)				
CHO	9.68 ± 1.57	17.85 ± 0.94*	16.6 ± 0.76*	13.88 ± 2.27
TG	0.79 ± 0.11	1.46 ± 0.13*,†	1.2 ± 0.15*,†	0.73 ± 0.13
HDL-C	4.49 ± 0.36	5.86 ± 0.19	5.96 ± 0.45	4.82 ± 0.68
LDL-C	0.65 ± 0.06	0.79 ± 0.08	1.19 ± 0.36	0.94 ± 0.17

* *P* < 0.05 *versus* non-DM control group.

† *P* < 0.05 *versus* DM-Ad-MIFi group. Data were mean ± SEM. CHO: Total Cholesterol; TG: Triglycerides; HDL-C: high-density lipoprotein cholesterol; LDL-C: low-density lipoprotein cholesterol.

### MIF gene silence inhibited atherosclerosis lesions

**Fig 3:**
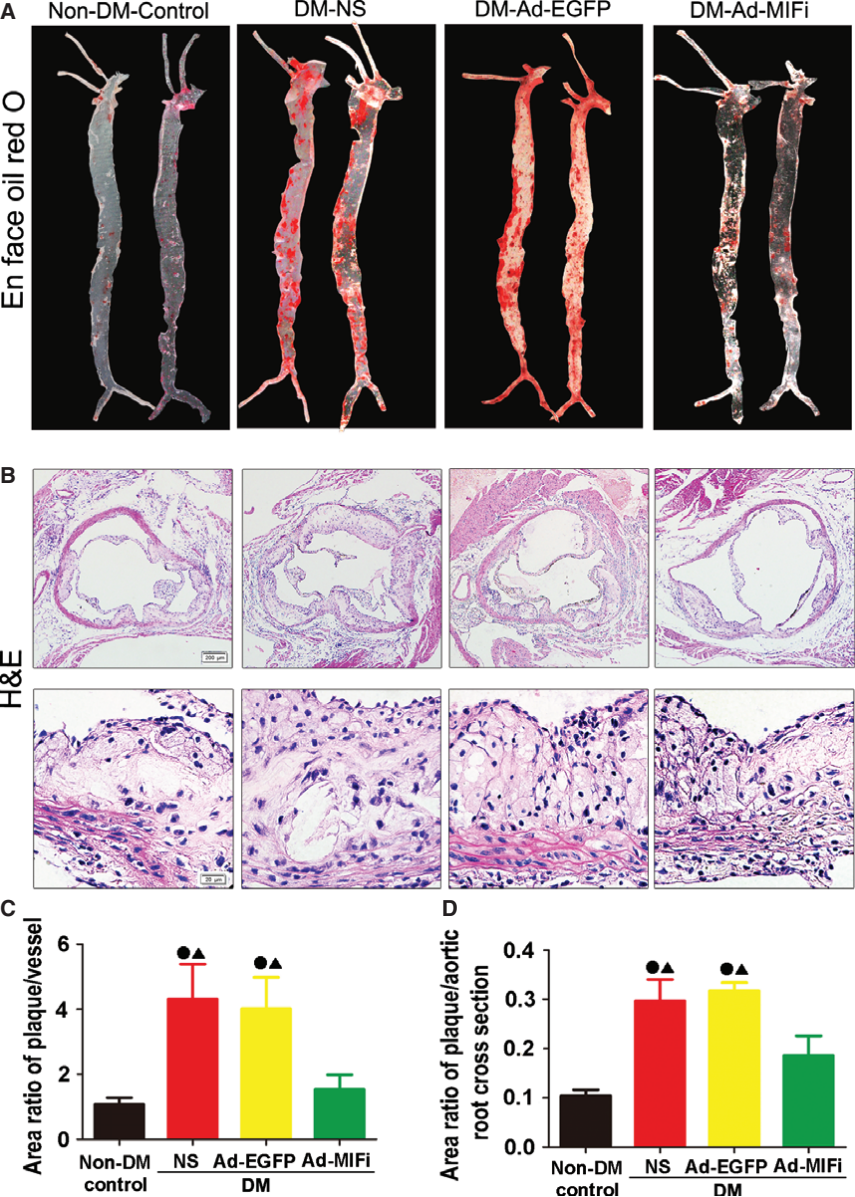
Pathology staining and quantitative analysis of atherosclerotic lesions in mice. (A) En face analysis of aortas. Atherosclerotic lesions were identified by Oil-Red-O staining. (B) Haematoxylin and eosin staining of aortic sinus cryosections (Top: scale bar = 200 μm, Bottom: scale bar = 20 μm). (C) The ratio of the atherosclerotic lesion area to the total vessel area, indicating level of atherogenesis. (D) The ratio of total atherosclerotic lesion area to aorta lumen area indicating mean size of atherosclerotic plaque. All quantitative data are means ± SEM (*n* = 10). •*P* < 0.05 *versus* non-DM-Control group and ▴*P* < 0.05 *versus* DM-Ad-MIFi group.

Under the condition of hyperglycaemia, the ratio of plaque area/vessel area was significantly increased in the arteries, including carotid artery, aortic root and abdominal aorta (Fig.3A and C). Compared with the non-diabetic apoE−/− mice, the DM apoE−/− mice also showed a larger burden plaque, accompanied by the remarkably increased ratio of plaque area/total cross-sectional vessel wall area of aortic root (Fig.3B and D). The total plaque area of aortae and the local AS lesions of aortic root in DM-Ad-MIFi group were significantly decreased in comparison with DM-Ad-EGFP group (Fig.3A–D). Thus, MIF gene interference significantly inhibited the AS lesions.

### MIF gene interference stabilized atheromatous plaque

**Fig 4:**
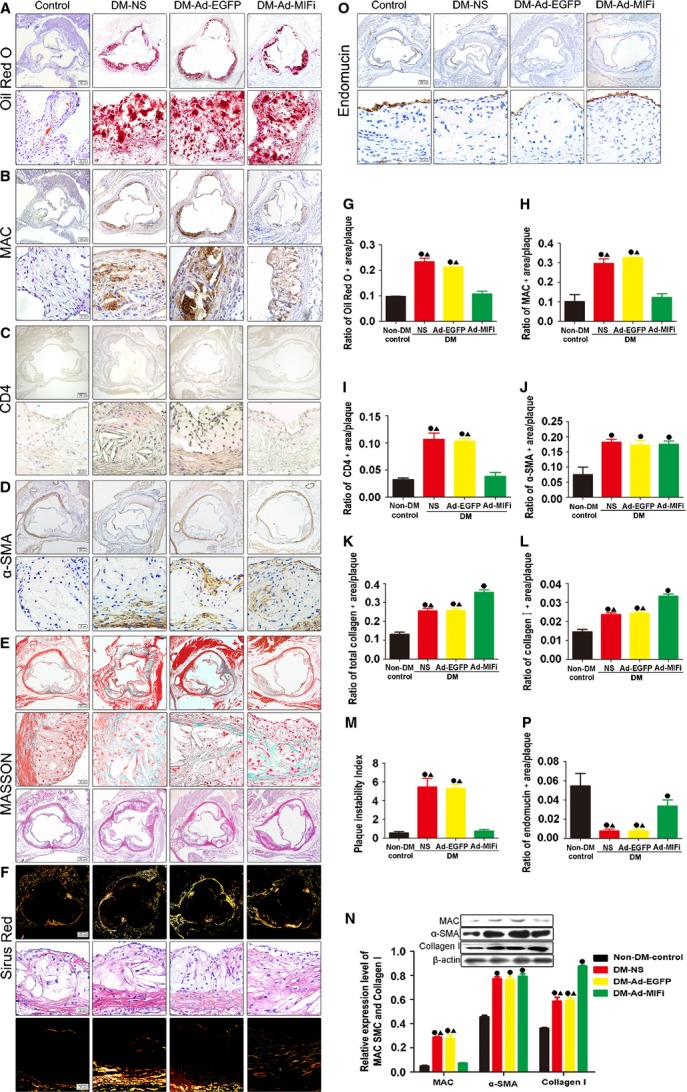
MIF gene interference stabilized atheromatous plaque. (A, D and E) Representative images by Oil-Red-O, Masson-trichrome and Sirius-red (for differentiation of collagen I and III) staining in atheroslerotic lesions of aortic root in Ad-MIFi, Ad-EGFP, NS control and non-DM control groups (*n* = 8, Top: scale bar = 200 μm; Bottom: scale bar = 20 μm). (B, C, D and P) Representative images by immunohistochemical staining for α-SMA of smooth muscle cells (SMCs), for MAC387 of macrophages, for CD4 of T cell and for endomucin of endothelial cells (ECs) (brown; Top: scale bar = 200 μm; bottom: scale bar = 20 μm). (G, H, I, J, K, L, and Q) Relative quantitative analysis of the contents of lipids, macrophages, CD4, SMCs, total collagen, collagen I and ECs (*n* = 7). (M) Quantitative analysis of plaque vulnerability index. (N) The ratio of collagen I to total collagen. (O) Western blots for MAC387, a-SMA, collagen I and β-actin in aorta (*n* = 8) and quantitative analysis. All quantitative data are means ± SEM. •*P* < 0.05 *versus* non-DM-Control group and ▴*P* < 0.05 *versus* DM-Ad-MIFi group.

Plaque stability chiefly depends on the contents of lipids, macrophages, SMCs and collagen, especially collagen I. Diabetic apoE−/−mice showed the increased lipid, macrophage, collagen I, III and SMCs, as detected by immunohistochemical staining. Thus, STZ-induced hyperglycaemia accelerated atherosclerosis process and vulnerability of atheromatous plaque in apoE−/− mice. However, the lipids, together with macrophages decreased, while the total collagen, even collagen I tended to be up-regulated in response to the lower level of MIF (Fig.4A, B, E, H and K). While there was no statistical difference in SMCs contents between Ad-MIFi group with Ad-EGFP group (Fig.4D and J). Also, MIF gene interference decreased plaque instability index and stabilized atheromatous plaque (Fig.4M). It is known that Masson staining can differentiate the total collagen, while Sirus red staining is special for differentiating the types of collagen. The percentage of collagen I in the total collagen also influences plaque stability. Our results revealed the ratio of collagen I/total collagen decreased after the MIF gene interference (Fig.4L). The protein levels of macrophage marker, SMCs marker and total collagen detected by western blot also showed similar changes (Fig.4N). MIF is a pleiotropic macrophage and T-cell cytokine. MIF gene interference also decreased T-cells infiltration in diabetic apoE−/− mice (Fig.4C and I). Thus, MIF gene interference could modify the plaque composition and increase vulnerable plaque stability. In addition, our immunohistochemical staining showed the accumulated endothelial cells (ECs) and the improved integrity of endothelium in the plaques of the DM-Ad-MIFi mice (Fig.4O and P).

### MIF gene interference improved lipid metabolism and glucose intolerance

**Fig 5:**
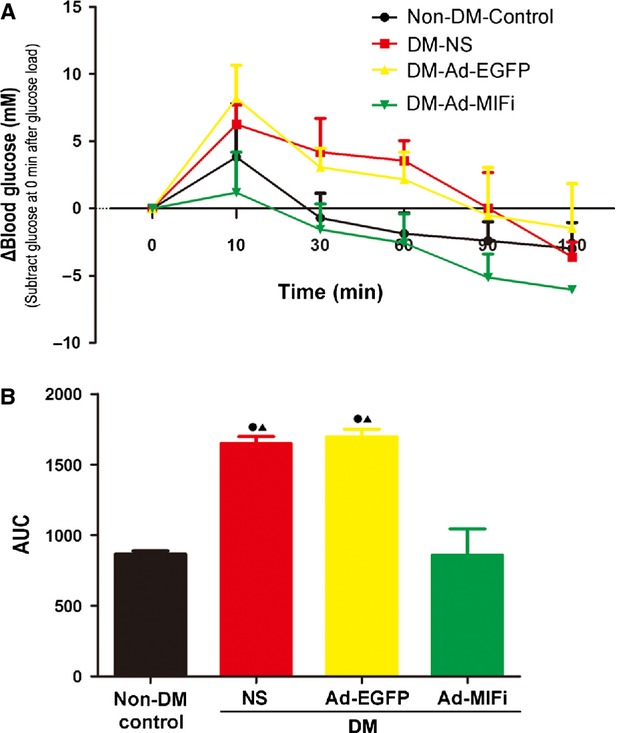
The intraperitoneal Glucose Tolerance Test (ipGTT) and its Area Under Curve analysis. (A) Glucose tolerance test in the four mice group (2 g of glucose per kg bodyweight intraperitoneal injection (i.p.) after a 6-hr fast in the daytime 3 days before euthanasia. Blood glucose levels were monitored at time-points indicated (*n* = 6). The data are revealed by subtracted the 0 min. glucose level. (B) The area under the curve (AUC) of ipGTT using the linear method of the trapezoid rule. AUC of ipGTT at different time-points was compared using one-way anova. All quantitative data are means ± SEM. •*P* < 0.05 *versus* non-DM-Control group and ▴*P* < 0.05 *versus* DM-Ad-MIFi group.

Bodyweight and biochemical parameters are described in Table1. Before the STZ injection, there were no significant differences in bodyweight, blood glucose, TG and TC levels among the four groups. All apoE−/− mice gradually lost bodyweight after the STZ injection. At the end of the study, the bodyweight of diabetic mice remained significantly lower than that of non-diabetic mice, which is consistent with the previous report 25. However, we did not find any effects of MIF gene interference on bodyweight of the diabetic mice, although it decreased blood glucose and TG levels. On the basis of it, we further performed a study on whether MIF has an effect on improvement of glucose intolerance of DM apoE−/− mice by GTT. In this experiment, the Figure5A revealed the blood glucose changes (that is the blood glucose level at different time-point subtracting that at the 0 min. after glucose load), while the Figure5B showed AUC corresponding to the Figure5A, and the larger AUC reflected the down-regulated glucose tolerance. A larger AUC was observed in the DM mice than that in non-DM apoE−/− mice (1469 ± 49.65 *versus* 864.75 ± 23.82, *P* < 0.05). The results demonstrated that a deterioration of glucose intolerance because of STZ-induced hyperglycaemia. While MIF gene interference could effectively improve glucose intolerance. We also detected a slight reduction in the TC level in Ad-MIFi group, while there were no statistical differences using one-way anova analysis, compared with the DM-Ad-EGFP group (*P* > 0.05). However, using paired samples *t*-test, there was a statistical reduction in the blood TC level in Ad-MIFi transfected apoE−/−mice with 23 week old, compared with those with 14 week old (*P* < 0.05) (Fig. S2, see Data S1). Thus, MIF gene interference might reduce blood TC to a certain extent. However, there was no statistical significance in blood LDL-C and HDL-C level during the experiment.

### MIF gene interference inhibited the expression of inflammatory cytokines: IL-6, TNF-α, VCAM1, ICAM1 and MCP-1

**Fig 6:**
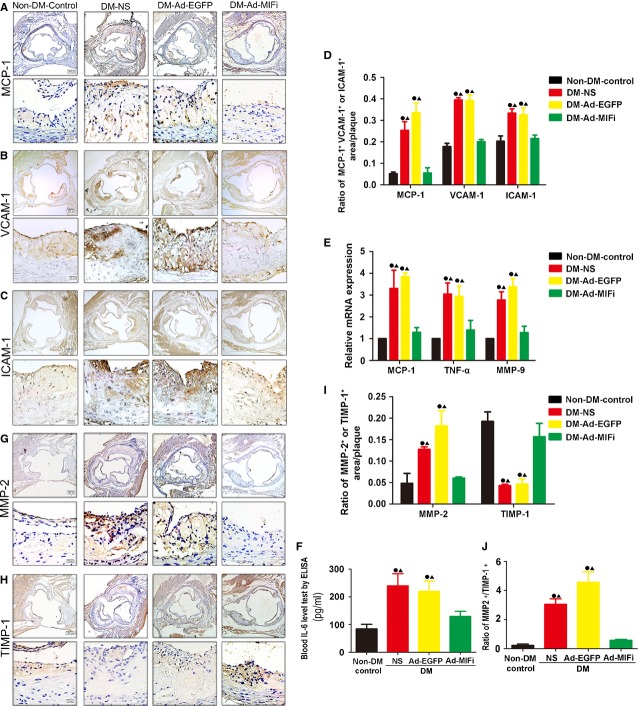
MIF gene interference attenuated the expression of inflammatory cytokine and matrix-related factor. (A, B, C, G and H) Representative images by immunohistochemical staining for MCP-1, VCAM1, ICAM-1, MMP-2 and TIMP-1 in atheroslerotic lesions of aortic root in Ad-MIFi, Ad-EGFP, NS control and non-DM control groups (*n* = 8, brown; Top: scale bar = 200 μm; Bottom: scale bar = 20 μm). (D and I) Quantitative analysis of the contents of MCP-1, VCAM1, ICAM-1, MMP-2 and TIMP-1 (*n* = 7). (E) Real-time PCR analysis of mRNA expression of MCP-1, TNF-α and MMP-9 (*n* = 8) in the vessels. (F) Blood IL-6 level tested by ELISA (*n* = 10). (G) The ratio of MMP-2/TIMP-1. All quantitative data are means ± SEM. •*P* < 0.05 *versus* non-DM-Control group and ▴*P* < 0.05 *versus* DM-Ad-MIFi group.

Interleukin-6, TNF-α, VCAM1, ICAM1 and MCP-1 are important inflammatory cytokines that are engaged in the development of atherosclerosis, which can exacerbate it. In this study, we evaluated IL-6, TNF-α, VCAM1, ICAM1 and MCP-1 expression in the four groups. The higher protein or mRNA level of VCAM1, ICAM1 and MCP-1 in diabetic mice was observed. MIF gene interference almost completely restored the protein level of VCAM1, ICAM1 and MCP-1 to normal level (Fig.6A–E). In addition, the serum IL-6 level was significantly higher in the diabetic mice (Fig.6F). Similarly, it was reduced after MIF gene interference. Moreover, MCP-1 and TNF-α mRNA expression in the aortae also showed consistent results (Fig.6E). Since VCAM1, ICAM1 and MCP-1 promotes macrophages recruitment and could be secreted by ECs and SMCs 26,27, the atheroclerotic plaque compositions in Ad-MIFi group may be attributed to the decreased VCAM1, ICAM1 and MCP-1 expression.

### MIF gene interference attenuated the expression of plaque matrix-related factor such as MMP-2, MMP-9 and TIMP-1

Immunohistochemical staining showed that exposure to high glucose could significantly decrease the expression of TIMP-1 and increase the expression of MMP-2 in aortic tissues of apoE−/− mice (Fig.6G–I). RT-PCR results showed the increased expression of MMP-9 mRNA in the aortas from diabetic mice (Fig.6E). However, immunohistochemical staining showed the reduced MMP-2 content but the increased TIMP-1 content in the Ad-MIFi group (Fig.6G–I). MMP-9 mRNA level was also reduced in the DM-Ad-MIFi group (Fig.6E). Interestingly MMP-2/TIMP-1 ratio, which is attributed to matrix composition in the plaque, showed a significant increase in the diabetic mice compared with the non-diabetic mice. Moreover, the decreased ratio of MMP-2/TIMP-1 was observed after MIF gene interference (Fig.6J).

### Increased MIF and CD74 expression in patients with diabetes and CAD

**Fig 7:**
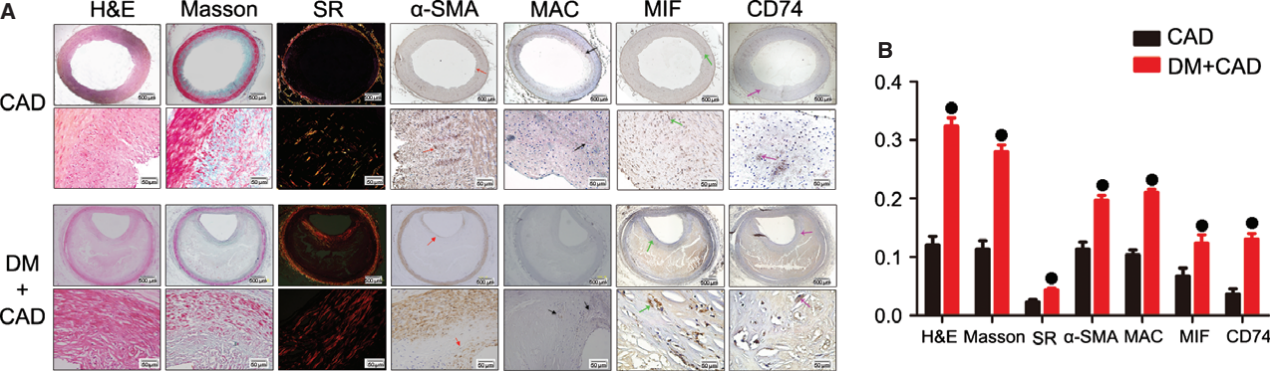
An increased expression of MIF and its ligand CD74 was detected in human left anterior descending branch from the patients with DM and CAD. (A) Representative images by haematoxylin and eosin, Masson and Sirius-red (SR) staining and by immunohistochemical staining for α-SMA, MAC387, MIF and CD74 in patients with CAD alone and the CAD patients with DM (*n* = 5/group; Top: scale bar = 500 μm; bottom: scale bar = 50 μm). Different colour arrows represent corresponding positive staining (Red: α-SMA; Black: MAC387; Green: MIF and Purple: CD74). (B) All quantitative data are means ± SEM. •*P* < 0.05 *versus* CAD group.

Immunohistochemical staining demonstrated that MIF was overexpressed in the LAD branch (LAD) from CAD patients with DM, compared with the patients with CAD alone (Fig.7). Moreover, CD74 expression was significantly ascended. Haematoxylin and eosin staining showed a larger plaque area in the patients with DM and CAD. The increased collagen and SMCs contents were also observed in human atheromatous plaques with CAD alone. The content of macrophages increased slightly in patients with CAD and DM, compared with that in patients with CAD alone. These results indicated that the larger and relatively vulnerable plaques existed in diabetic atherosclerosis patients, compared with the patients with CAD.

## Discussion

The key finding of this study is that MIF gene interference inhibits the development of atherosclerotic lesions and increases atheromatous plaque stability in STZ-induced diabetic apoE−/− mice by decreasing macrophages and lipids deposition of plaque. The antiatherosclerotic effect of MIF gene interference likely results from the decreased production of circulating and local inflammatory cytokines, improved glucose intolerance, down-regulated TC level, increased collagen I/III contents, decreased ratio of MMP-2/TIMP-1 and plaque instability index. The underlying mechanism involves inflammation inhibition in STZ-induced diabetes-associated atherosclerosis.

Mice deficient in apoE develop severe atherosclerosis on a 4.5% fat-containing diet, become a powerful tool in atherosclerosis research. STZ-induced diabetic apoE−/− model has previously been confirmed as feasible for the study of diabetes-associated atherosclerosis by the Animal Models of Diabetic Complications Consonium (AMDCC) 28 and some previous studies 29. The recommendation dose of STZ by AMDCC is low-dose strategy (40–50 mg/kg/day for 5 days) compared with the high-dose STZ (150–200 mg/kg once), which can minimizes non-specific toxic effect and provide a robust and consistent hyperglycaemic response 28. When diabetes is induced in apoE-deficient, a marked increase in plasma cholesterol levels is observed in diabetic mice compared to non-diabetic controls fed the same diet. Moreover, there is no difference in aggravated atherosclerotic lesions between diabetic mice induced by STZ and clinical diabetic patients 25. In this study, only slightly toxic effect from STZ was detected in liver of mice after intraperitoneal injecting of low-dose STZ for 5 days. In addition, the increased cell apoptosis were detected in the STZ-induced diabetic-atherosclerotic plaque, compared with that of non-diabetic mice (*P* < 0.05; Fig. S3, see Data S1).

Migration inhibitory factor, one of the earliest cytokines discovered, inhibits the random migration of peritoneal macrophages. Previous findings that blockade of MIF with a neutralizing MIF antibody markedly reduces neointimal macrophages, less foam cell accumulation, and increases quantity of SMCs and amount of collagen in the intima but no difference in intima thickening in atheromatous lesion after vascular injury in apoE−/− mice, which suggest a role of MIF in destabilization of atheromatous plaques 11. In contrast, Chen *et al*. observed that immunoneutralization of MIF reduced inflammation, impaired cellular proliferation, and decreased the intima thickening in carotid arteries of LDLr−/− mice injured in another manner 10. In this study, we have identified MIF as an important regulator of the cellular composition in the diabetic apoE−/− mice model. MIF gene interference reduced lipids, macrophages, and T cells, while increased collagen and ECs in diabetic associated atheromatous lesion, which could increase stability of the vulnerable plaque. However, we did not detect significant changes of SMCs contents after MIF gene interference. Previous study has found that MIF is expressed in human vascular ECs, SMCs and macrophages. Schober, *et al*. 11 has further confirmed MIF was expressed in ECs in apoE−/− mice fed an atherogenic diet. Our results have demonstrated that the ECs content and the integrality of endothelium in the Ad-MIFi group were significantly improved. Also, our current data indicate a similarity in the cellular pattern of MIF expression in the diabetic apoE−/− mice model and human coronary artery from CAD patients with DM. Hence, our study has further demonstrated that MIF gene interference effectively inhibits atherosclerosis process and stabilizes vulnerable plaque in STZ-induced diabetic apoE−/− mice.

Serum cholesterol rose in STZ-induced diabetic apoE−/− mice that consumed the chow diet. However, the effects of MIF on TG and TC still remain controversial. Pan *et al*. also found deficiency of MIF reduced TC, LDL and TG, but not HDL levels 15. While Schober *et al*. found TC and TG levels did not differ between MIF mAb-treated and isotype control-treated apoE−/− mice 11. Similar results were observed in LDLR−/− mice 25. This discrepancy could result from differences in the animal models or from incomplete tissue MIF neutralization or knockdown. Our study found MIF gene interference reduced blood TC and TG to a certain extent in diabetic apoE−/− mice. However, to date, it is unclear how MIF gene interference affects TG and TC. In this study, we checked mRNA levels of PPARα and LXRα genes in the liver, chiefly regulating lipid metabolism and there is no significant difference among DM-NS, DM-Ad-EGFP and Ad-MIFi-transfected mice fed on chow diet (*P* < 0.05; Table S3 and Fig. S4, see Data S1).

Previous study has shown that blood TC level is not the solo reason for the aggravation of AS, and deterioration of glucose intolerance is also involved in the process of atherosclerosis 28. Our results have shown deterioration of glucose intolerance in STZ-induced diabetic apoE−/− mice. We also have provided direct evidence that the pro-inflammatory cytokine MIF plays a key role in glucose homoeostasis. MIF gene interference is linked to impaired glucose tolerance, and lower blood glucose level in diabetic apoE−/− mice. Atsumi *et al*. 13 confirms that MIF could act in an autocrine/paracrine manner to regulate glucose metabolism by stimulating glucose uptake and glucose catabolism in peripheral tissue. These data provide the evidence that MIF is not only an associated bystander linked to glucose metabolism but can also be a key causative cytokine in the development of metabolic abnormalities. While, its role and mechanism of action remains to be further characterized. In this study, MIF gene interference improves glucose intolerance, which is likely attributed to the improvement of pancreas function or insulin related receptor. Thus, MIF appears as a potential target for intervention in various glucose metabolism abnormalities, including type 2 DM.

The profile of activities of MIF *in vivo* and *in vitro* is strongly suggestive of a role for MIF in the pathogenesis of many inflammatory diseases, including atherosclerosis, and hence antagonism of MIF is suggested as a potential therapeutic strategy in inflammatory disease. The proinflammatory cytokine MIF is an essential, upstream component of the inflammatory cascade and influences the effects of TNF-α and IL-6 12,17. IL-6 has been identified as a risk factor for CAD. Using rIL-6 treatment increases lesion size in C57BL/6 and apoE deficient mice, while IL-6−/−/apoE−/− double knockout mice results in the enhanced atherosclerotic lesion formation 30,31. Our study has showed STZ-induced diabetic apoE−/− mice have significantly increased plasma MIF and IL-6 level. The significant higher TNF-α, VCAM1, ICAM1 and MCP-1 mRNA levels were also detected in diabetic associated atheromatous lesion, which further supports on the increased inflammation status in STZ-induced diabetic mice. However, MIF gene interference notably inhibits inflammation by reducing the local or circulating level of cytokines, suh as MIF, IL-6, TNF-α, VCAM1, ICAM1 and MCP-1 in diabetic apoE−/− mice. In the process of atherogenesis, widely accepted as a chronic inflammatory disease, MIF production is not restricted to immune cells, such as macrophages and lymphocytes. Vascular ECs and SMCs, were also found to produce MIF after inflammatory stimulation 32. In macrophages, MIF induces secretion of TNF-α, nitric oxide, IL-1β and IL-8 which are angiogenic and inflammatory mediators with abundant presence in complicated atherosclerotic lesions 33. Some cellular elements of the arterial wall, like ECs and SMCs also could secret MCP-1 27,34. MCP-1 promotes monocytes recruitment, which could also stimulate T cell to enter into vascular lesions 12. Monocytes together with T cells are engaged in the formation of atherosclerotic lesions as well as in human advanced plaques 16. Our study also showed MIF gene interference decreased T-cells infiltration in diabetic apoE−/− mice. Thus, MIF gene interference inhibits inflammation, which could chiefly attribute to its inhibiting of atherosclerosis process and stabilizing of atherosclerosis plaque.

Both inflammation and MMPs play a critical role in the progress of vulnerable atherosclerotic plaques 35. MIF is a potentially important upstream activator of MMP system. Modulation of inflammatory process and MMPs may be a potential molecular basis of the MIF-mediated plaque regression and plaque-stabilizing activity 36. Accumulating clinical and experimental evidence have shown that MMP-2 and MMP-9 content and activity are correlated with atherosclerosis development, since they could decrease basement membrane and promote SMCs migration and proliferation 37. Our study has further confirmed that MIF gene interference could decrease MMP-2 content in aortic tissues. TIMP-1 is a glycoprotein that could inhibit the activity of MMPs, especially MMP-1, MMP-2 and MMP-9 35. The MMP-9 content is increased in the unstable human carotid plaques 38. The equilibrium between MMPs and TIMPs determines the net proteolytic activity of the degradation enzymes and their consequences. Previous studies have shown a countered relationship between atherosclerosis and TIMPs, suggesting TIMP-1 is up-regulated in fibrous plaques 39. Overexpression of TIMP-1 expression results in reduction in plaque area in apoE−/− mice and ease of aortic aneurysm degeneration and rupture in rat model 41,42. In contrast, deficiency of TIMP-1 expression significantly increases the matrix degradation associated with atherosclerotic lesions in apoE−/− mice. TIMP3, a different member of the TIMPs family, the endogenous inhibitor of A disintegrin and metalloprotease domain 17 (ADAM17) and others MMPs, as a gene modifier for insulin resistance and vascular inflammation in mice has recently been identified 44. To date, the relationship between MIF and TIMPs in diabetic atherosclerosis remains unclear. In our diabetic mice, TIMP-1 expression is decreased after MIF gene interference. Importantly, the ratio of MMP-2/TIMP-1 remains statistical significance after Ad-MIFi gene therapy. Our study has also demonstrated that MIF, MMP-2, MMP-9, TIMP-1 are involved in vulnerable atheromatous plaques in diabetic apoE−/− mice. Thus, MIF gene interference stabilizes atherosclerosis plaque likely by inhibiting MMP-2 and MMP-9 expression, up-regulating TIMP-1 expression and decreasing the ratio of MMP-2/TIMP-1. These results further supports MMPs/TIMP-1 may be a potential molecular basis of the MIF-mediated plaque-stabilizing activity in STZ-induced diabetic apoE−/− mice.

Intracellular functions of MIF have been reported previously 44,45. However, the intracellular targets of MIF in atherosclerotic lesion had not been studied in detail and the receptor-mediating MIF activity has not been clearly defined. Our study has showed the consistent changes between MIF and CD74 in DM apoE−/− mice and in human coronary artery from CAD patients with DM. CD74 is the cell surface form of class II invariant chain (Ii), interacts with MIF. In T cells, MIF-mediated activation of the JNK pathway leads to up-regulated gene expression of the inflammatory chemokine CXCL8. Activation of JNK signalling by MIF involves the upstream kinases PI3K and SRC and is found to be dependent on CXCR4 and CD74 46. Bernhagen *et al*. have identified that the archaic cytokine MIF as a non-canonical ligand of the CXC chemokine receptors CXCR2 and CXCR4 in inflammatory and atherogenic cell recruitment 47. In atherogenic monocyte recruitment, MIF-induced monocyte adhesion involves CD74 and CXCR2, which form a signalling receptor complex. Previous study has also shown that high glucose level could contribute to the increased CD74 expression in DM patients' podocyte and tubular cell 48. The serum MIF concentration is elevated in T2DM individuals 19 and MIF and CD74 are two overexpressed genes in human diabetic nephropathy. Endocytosed or endogenous MIF interacts with Jun activation domain-binding protein 1, originally described as transcriptional co-activator for the transcription factor AP-1 49. However, we have not found any effect of MIF gene interference on the expression of Jab1 in the diabetic apoE−/− mice.

There are several limitations of our study that must be acknowledged. First, being hard to obtain enough blood from the mice, we did not further investigate the changes of glycated haemoglobins and insulins, although blood glucose and ipGTT were observed in this study. On the other hand, we have mainly focused attention on attenuation of atherosclerotic lesions in STZ-induced diabetic apoE−/− mice using gene silencing of MIF. Although plaque stability is involved in this study, mouse atherosclerotic lesion rupture has not been observed in detail. In addition, the regulatory mechanism of CD74 and Jab-1 expression has not been well-investigated. IPGTT data strength is also limited by the size of the sample. Although recent study showed that lack of TIMP3 increases inflammation and polarizes macrophages towards a more inflammatory phenotype resulting in increased atherosclerosis 50,51, the relationship between TIMP3 and MIF did not be detected in our study. Finally, the use of MIF knockout mice would have strengthened our results.

In conclusion, our study has demonstrated that MIF gene interference is able to inhibit atherosclerotic lesions and increase plaque stability in STZ-induced diabetic apoE−/− mice. MIF inhibition could be a novel and promising approach to the treatment of DM-associated AS.

## Supporting Information

**Figure S1** Haematoxylin and eosin staining of serial tissue sections of liver.

**Figure S2** Plasma total cholesterol level in mice.

**Figure S3** Apoptotic cells in diabetic-atherosclerotic plaque.

**Figure S4** Real-time PCR was performed to determine expression of genes regulating lipid metabolism (PPARα and LXRα) in the liver.

**Data S1** Methods for detection apoptosis and mRNA levels PPARα and LXRα in the liver and Tables for mRNAi sequence of NPR-C-site and primers for quantitative reverse transcriptase-polymerase chain reaction.
