# Supplementary figures and images for: Attenuation of atherosclerotic lesions in diabetic apolipoprotein E-deficient mice using gene silencing of macrophage migration inhibitory factor
Source: J Cell Mol Med. 2015 Feb 8;19(4):836–49. doi: 10.1111/jcmm.12521 (PMC4395198; doi:10.1111/jcmm.12521)

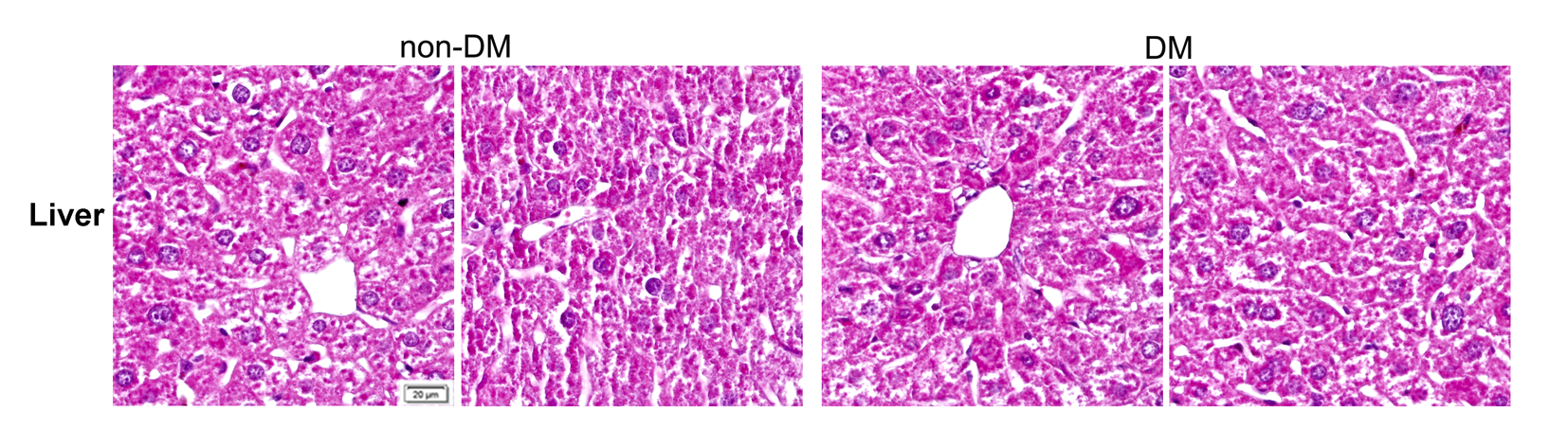

Supplement: Supplementary file 1 [file jcmm0019-0836-sd1.tif]

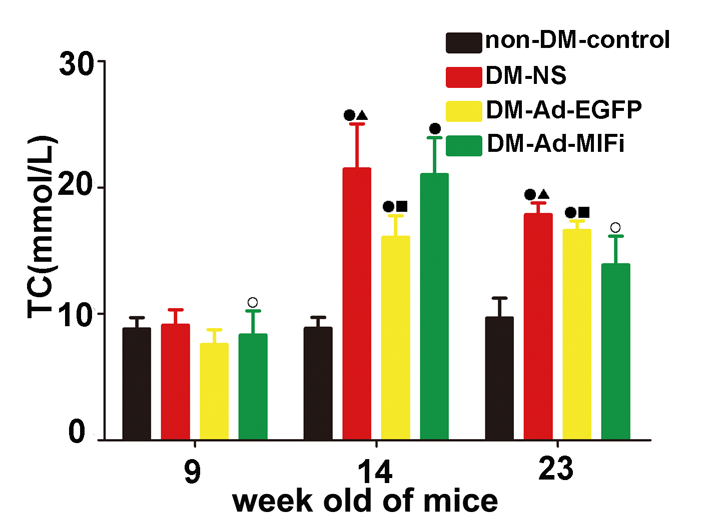

Supplement: Supplementary file 2 [file jcmm0019-0836-sd2.tif]

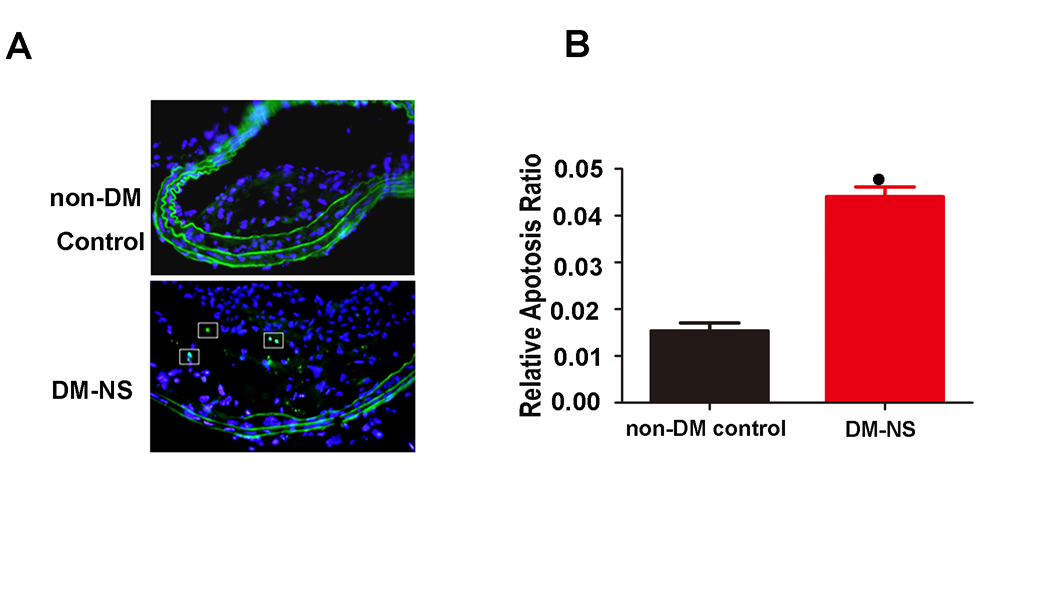

Supplement: Supplementary file 3 [file jcmm0019-0836-sd3.tif]

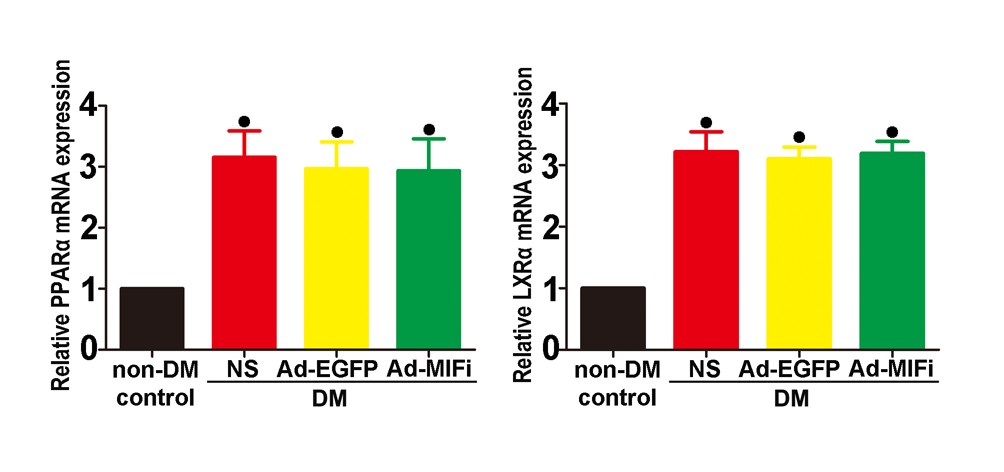

Supplement: Supplementary file 4 [file jcmm0019-0836-sd4.tif]
