# Supplementary material for: Attenuation of atherosclerotic lesions in diabetic apolipoprotein E-deficient mice using gene silencing of macrophage migration inhibitory factor
Source: J Cell Mol Med. 2015 Feb 8;19(4):836–49. doi: 10.1111/jcmm.12521 (PMC4395198; doi:10.1111/jcmm.12521)
Supplement: Supplementary file 5 [file jcmm0019-0836-sd5.doc]

**Supplemental Material**

**Supplemental Methods**

**Histochemical studies**

Apoptotic cells in atherosclerotic plaques were detected using terminal deoxyribonucleotide transferase (TdT)-mediated nick-end labeling (TUNEL) staining using an in situ apoptosis detection kit (TACS, Trevigen). The sections were counterstained with hematoxylin for 30 seconds (pale blue nuclei). The number of apoptotic cells was counted from TUNEL-stained specimens.

**Quantitative real-time PCR**

Real-time PCR was performed to determine mRNA levels of genes regulating lipid metabolism (PPARα and LXRα) in the liver by using SYBR Green Technology (Bio-Rad, USA), and the mouse housekeeping gene β-actin was applied as an internal control. The sequences of primers for target gene sequence were mentioned on *Supplementary Table III.* The data were analyzed by the 2^−△△CT^ method. All experiments were repeated for at least three times.

**Supplemental Tables**

**Table I. mRNAi sequence of NPR-C-site** A, B, C, D ,and negative control

| Site | Forward primer (5’-3’) | Reverse primer (5’-3’) | RNAi effeciency (%) in vitro |
| --- | --- | --- | --- |
| Negative control | tgctgAAATGTACTGCGCGTGGAGACGTTTTGGCCACTGACTGACGTCTCCACGCAGTACATTT | cctgAAATGTACTGCGTGGAGACGTCAGTCAGTGGCCAAAACGTCTCCACGCGCAGTACATTTc | 0 |
| Site A | TGCTGAACATTGGTGTTCACGATGAAGTTTTGGCCACTGACTGACTTCATCGTACACCAATGTT | CCTGAACATTGGTGTACGATGAAGTCAGTCAGTGGCCAAAACTTCATCGTGAACACCAATGTTC | 90 |
| Site B | TGCTGCCACACAGCAGCTTACTGTAGGTTTTGGCCACTGACTGACCTACAGTACTGCTGTGTGG | CCTGCCACACAGCAGTACTGTAGGTCAGTCAGTGGCCAAAACCTACAGTAAGCTGCTGTGTGGC | 29 |
| Site C | TGCTGAATAGTTGATGTAGACCCGGTGTTTTGGCCACTGACTGACACCGGGTCCATCAACTATT | CCTGAATAGTTGATGGACCCGGTGTCAGTCAGTGGCCAAAACACCGGGTCTACATCAACTATTC | 42 |
| Site D | TGCTGTAATAGTTGATGTAGACCCGGGTTTTGGCCACTGACTGACCCGGGTCTATCAACTATTA | CCTGTAATAGTTGATAGACCCGGGTCAGTCAGTGGCCAAAACCCGGGTCTACATCAACTATTAC | 71 |

**Table II.** Primers for quantitative reverse transcriptase-polymerase chain reaction

| Gene | Forward | Reverse |
| --- | --- | --- |
| MIF | 5’-CCCAGAACCGCAACTACA-3’ | 5’-GAGCGAGGCTCAAAGAAC-3’ |
| CD74 | 5’-AGATGCGGATGGCTACTCC-3’ | 5’-TCATGTTGCCGTACTTGGTAAC-3’ |
| MCP-1 | 5’-TTAAAAACCTGGATCGGAACCAA-3’ | 5’-GCATTAGCTTCAGATTTACGGGT-3’ |
| MMP-9 | 5’-CTGGACAGCCAGACACTAAAG-3’ | 5’-CTCGCGGCAAGTCTTCAGAG-3’ |
| TNF-α | 5’-AAGCAAGCAGCCAACCAG-3’ | 5’-TCTTCTGCCAGTTCCACG-3’ |
| Jab-1 | 5’-GCTTCCGGGAGTGGTATGG-3’ | 5’-CGCCGCCAGGATTTCTTGT-3’ |
| β-actin | 5’-GTGACGTTGACATCCGTAAAGA-3’ | 5’-GCCGGACTCATCGTACTCC-3’ |

MIF, macrophage migration inhibitory factor; MCP-1, monocyte chemoattractant protein-1; MMP-9, matrix metalloproteinase-9; TNF-α, tumor necrosis factor-α.

**Table III. Primers for quantitative reverse transcriptase-**polymerase chain reaction

| Gene | Forward | Reverse |
| --- | --- | --- |
| PPARα | 5’-TCTGTGGGCTCACTGTTCT-3’ | 5’-AGGGCTCATCCTGTCTTTG-3’ |
| LXRα | 5’-GGATAGGGTTGGAGTCAGCA-3’ | 5’-CTTGCCGCTTCAGTTTCTTC-3’ |

PPARα, peroxisome Proliferator-activated receptor α; LXRα,Liver X receptor α.

**Legends to the supplemental figures**

**Figure I.** H&E staining of serial tissue sections of liver. Compared with non-diabetic apoE-/-mice, the slight cellular edema, cytoplasm puffing and steatosis of hepatocyte were observed in diabetic mice.

**Figure II.** Plasma total cholesterol level in mice. After STZ injection, diabetic mice showed significant higher total cholesterol (TC) than the non-DM mice. At the end of the study, there was no difference between Ad-MIFi group and the non-DM group (*n=8*). All quantitative data are means ± SEM. ●*P<0.05* *versus* the same time point control group, ▲*P<0.05* *versus* the 9 week DM-NS group, ■*P<0.05* *versus* the 9 week DM-Ad-EGFP group and ○*P<0.05* versus the 14 week DM-Ad-MIFi group.

**Figure III.** Apoptotic cells in diabetic-atherosclerotic plaque. After STZ injection, diabetic mice showed significant increased apoptotic cells than the non-DM mice. All quantitative data are means ± SEM. ●*P<0.05 versus* non-DM-Control group.

**Figure IV.** Real-time PCR was performed to determine expression of genes regulating lipid metabolism (PPARα and LXRα) in the liver. The result showed there is no significant difference among DM-NS, DM-AdEGFP and DM-Ad-MIFi group. All quantitative data are means ± SEM. ●*P<0.05 versus* non-DM-Control group.
